# Musculature of an Early Cambrian cycloneuralian animal

**DOI:** 10.1098/rspb.2023.1803

**Published:** 2023-10-11

**Authors:** Huaqiao Zhang, Shuhai Xiao, Mats E. Eriksson, Baichuan Duan, Andreas Maas

**Affiliations:** ^1^ State Key Laboratory of Palaeobiology and Stratigraphy, Nanjing Institute of Geology and Palaeontology, Chinese Academy of Sciences, Nanjing 210008, People's Republic of China; ^2^ Department of Geosciences, Virginia Tech, Blacksburg, VA 24061, USA; ^3^ Department of Geology, Lund University, Lund 22362, Sweden; ^4^ Key Laboratory of Marine Geology and Metallogeny, First Institute of Oceanography, Ministry of Natural Resource, Qingdao 266061, People's Republic of China; ^5^ Galgenackerweg 25, Blaustein 89134, Germany

**Keywords:** introvert musculature, Priapulida, Scalidophora, Cycloneuralia, Cambrian Fortunian, South China

## Abstract

Cycloneuralians are ecdysozoans with a fossil record extending to the Early Cambrian Fortunian Age and represented mostly by cuticular integuments. However, internal anatomies of Fortunian cycloneuralians are virtually unknown, hampering our understanding of their functional morphology and phylogenetic relationships. Here we report the exceptional preservation of cycloneuralian introvert musculature in Fortunian rocks of South China. The musculature consists of an introvert body-wall muscular grid of four circular and 36 radially arranged longitudinal muscle bundles, as well as an introvert circular muscle associated with 19 roughly radially arranged, short retractors. Collectively, these features support at least a scalidophoran affinity, and the absence of muscles associated with a mouth cone and scalids further indicates a priapulan affinity. As in modern scalidophorans, the fossil musculature, and particularly the introvert circular muscle retractors, may have controlled introvert inversion and facilitated locomotion and feeding. This work supports the evolution of scalidophoran-like or priapulan-like introvert musculature in cycloneuralians at the beginning of the Cambrian Period.

## 1. Introduction

Ecdysozoa [1] is the most diverse taxon within the Bilateria [2,3]. It contains the Scalidophora (Kinorhyncha, Loricifera and Priapulida), Nematoida (Nematoda and Nematomorpha), and Panarthropoda (Tardigrada, Onychophora and Arthropoda) [3], with the former two constituting the Cycloneuralia [3], the monophyly of which is debated [4]. Molecular clock estimates indicate that the Ecdysozoa may have diverged in the Ediacaran Period [5], but it was not until the earliest Cambrian Period (*ca* 538.8 Ma [6]) when ecdysozoans first appeared in the fossil record, as represented by *Treptichnus pedum*, a putative priapulan trace fossil [7,8]. Unambiguous ecdysozoan body fossils, however, first appeared in the Early Fortunian Age (*ca* 536.4–531.8 Ma [6]) [9]. Indeed, a relatively abundant and diverse assemblage of ecdysozoan body fossils have been recovered from the Early Fortunian, as represented by the total-group ecdysozoan taxon *Saccorhytus* [10] and several crown-group cycloneuralian species [11].

Although the Fortunian ecdysozoan fossils are relatively abundant, their preservation is limited to cuticular integuments [10,12], with no labile internal tissues (e.g. muscles or nerve tissues) preserved—a preservational style known as Orsten-type preservation [13]. As a consequence, their phylogenetic interpretations are based exclusively on cuticular structures [10], and cannot be independently tested using soft-tissue anatomical features.

Here we report three-dimensionally phosphatized microfossils from the Fortunian Zhangjiagou section [10,14] in southern Shaanxi, China. Using scanning electron microscopy (SEM) and micro-CT, we demonstrate that these fossils preserve an introvert musculature system that consists of circular, longitudinal and radial muscles. The musculature system indicates a phylogenetic relationship with scalidophorans and possibly priapulans.

## 2. Material and methods

### (a) Material

The studied specimens were recovered from the Kuanchuanpu Formation at Zhangjiagou section [14], southern Shaanxi Province, China (electronic supplementary material, figure S1). The fossil-yielding bed (electronic supplementary material, figure S1c) has also been the focus of several previous studies [9,12,14], and it has been known to contain abundant ecdysozoans, including the crown-group cycloneuralians [9,12,15] and the total-group ecdysozoan *Saccorhytus* [10]. This bed falls within the small shelly fossil *Anabarites trisulcatus*-*Protohertzina anabarica* Assemblage Zone [16], which is considered to be Early Fortunian in age and about 536.4–531.8 Ma [6]. Rock samples from the Zhangjiagou section were macerated using diluted acetic acid (10%), and microfossils were handpicked from the residues under a binocular microscope. The studied specimens are deposited at the Nanjing Institute of Geology and Palaeontology (NIGP), Chinese Academy of Sciences (CAS), with accession numbers NIGP179459–179461.

### (b) Scanning electron microscopy

Selected specimens were glued to an aluminium stub for observation under a Hitachi SU3500 SEM. One of the specimens, NIGP179459, was subsequently selected for micro-CT scanning (see below). This specimen was then transferred back to an aluminium stub, coated with gold and observed at high magnification for studies of nanocrystals under a field-emission SEM TESCAN MAIA3.

### (c) Micro-CT scanning

Micro-CT scanning was conducted at the micro-CT lab at the NIGP, CAS, using a Zeiss Xradia 520 Versa instrument. We used a 50 kV operating voltage of the X-ray tube, with a thin filter (LE2) to avoid beam-hardening artefacts. Because of the microscopic size of specimen NIGP179459, a charge-coupled device (CCD)-based optical microscope (4×) was applied. This system produces datasets with a voxel dimension of 1.63 µm. We obtained 2501 equi-angular projections over 360°. The exposure time for each projection was one second. The volume data were processed using AVIZO (www.thermofisher.com/avizo/software) to produce volume renditions, slice movies, and virtual sections. Micro-CT scan data are reposited at the Science Data Bank (https://doi.org/10.57760/sciencedb.11228 [17]).

## 3. Results

### (a) Anatomy of NIGP179459

**Figure 1.**
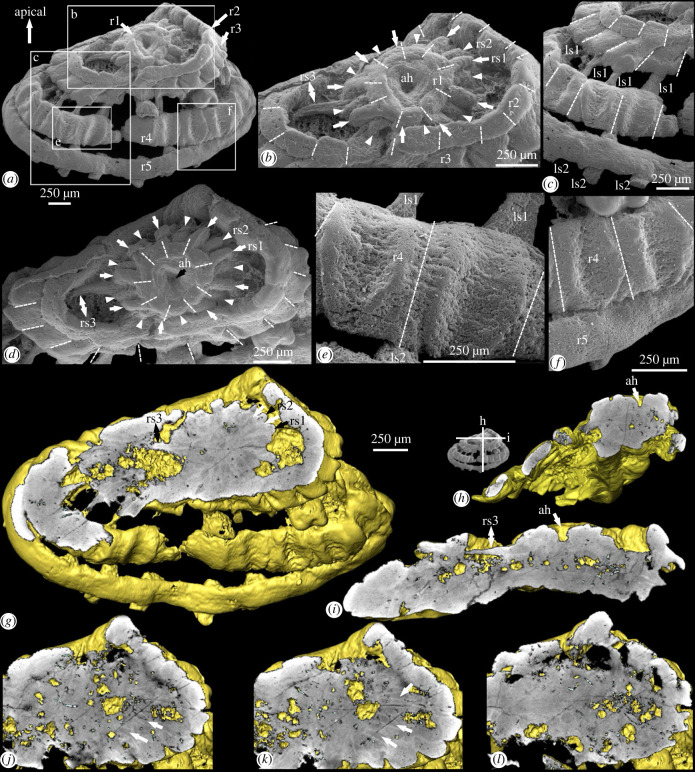
Anatomy of specimen NIGP179459. (*a–f*) SEM images; (*g–l*) micro-CT images based on volume rendition. (*a*) Apical–lateral view, boxed areas magnified in (*b*,*c*,*e,f*); (*b*) close-up of (*a*), showing apical end; (*c*) close-up of (*a*), showing localized gaps; (*d*) apical view, tilted about 35° from (*b*); (*e,f*) close-up views of (*a*), showing vertebra-like structures; (*g*) virtual section perpendicular to apical–abapical axis and through radial structures; (*h*,*i*) two orthogonal virtual sections parallel to the apical–abapical axis and through the apical hole, as denoted in SEM image in (*h*); (*j–l*) abapically successive virtual sections parallel to that in (*g*) and through radial structures. In (*b,d*), arrows and arrowheads denote the first and second circlets of radial structures. In (*b,d,g,i*), double-headed arrows denote the third ‘circlet’ of a single radial structure. In (*b–f*), dashed lines denote lobes on first and second, corrugations on the third, and vertebra-like structures on the fourth rings. In (*g,j,k*), white arrows denote grey stripes between radial structures. Abbreviations: ah, apical hole; ls1–2, first/second circlet of longitudinal structures; r1–5, first to fifth ring; rs1–3, first to third circlet of radial structures. Scale bar to the right of (*g*) applies to (*g–l*).

**Figure 2.**
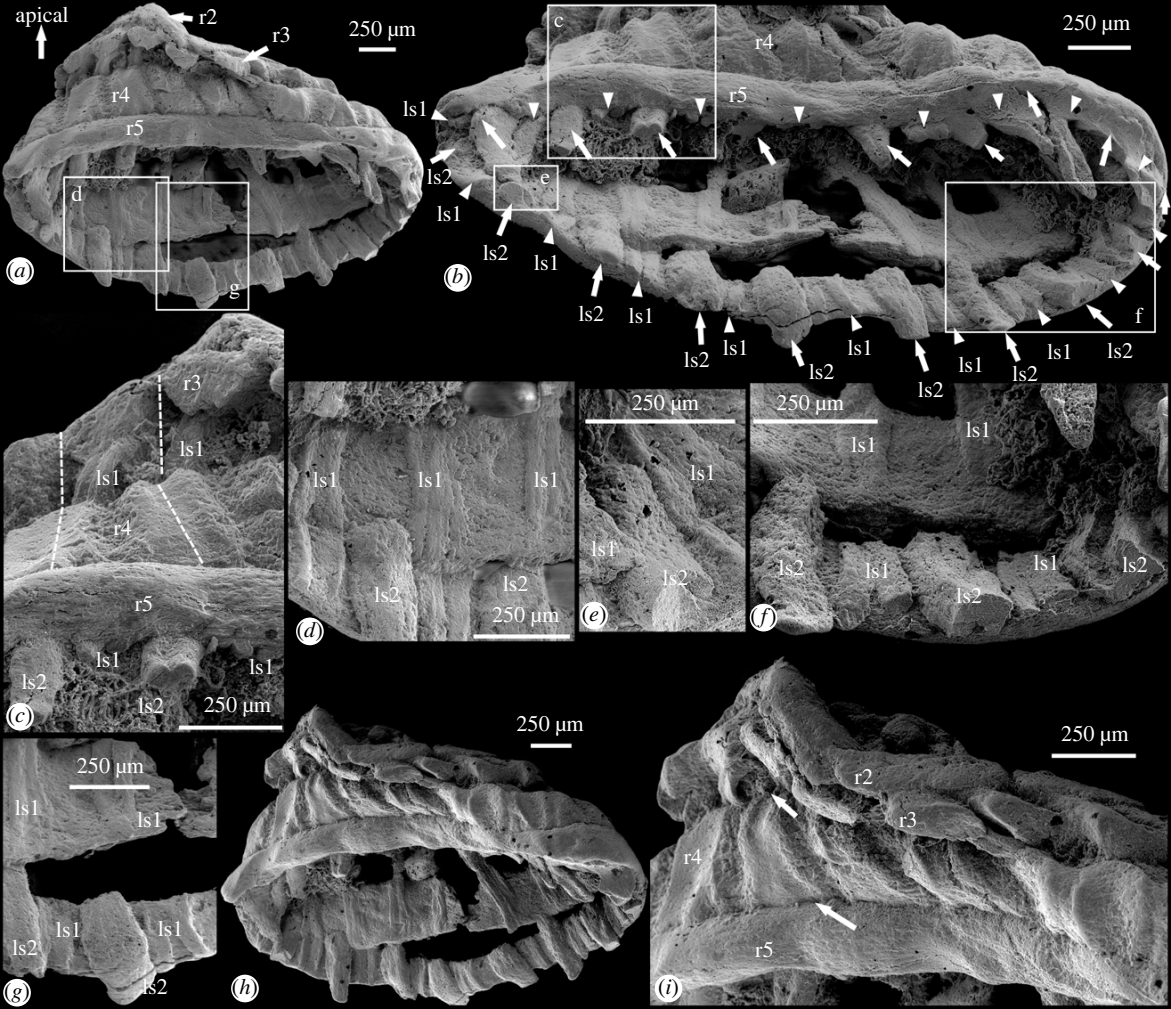
Anatomy of specimen NIGP179459. (*a*) Opposite side of the specimen in figure 1*a*, boxed areas magnified in (*d*) and (*g*); (*b*) close-up of abapical end, tilted about 40° from (*a*), with arrowheads and arrows denoting the first and second circlets of longitudinal structures, boxed areas magnified in (*c*,*e*,*f*); (*c*) close-up of (*b*), with dashed lines demarcating corrugations on the third and vertebra-like structures on the fourth rings; (*d*) close-up of (*a*), showing fibrous fabrics of longitudinal structures; (*e,f*) close-up views of (*b*), showing the second circlet of longitudinal structures restricted to the fifth ring; (*g*) close-up of (*a*), showing fibrous and bifurcated longitudinal structures; (*h*) view tilted about 30° from (*a*); (*i*) close-up of (*h*), arrows denoting the third to fifth rings that are compressed against each other. Abbreviations as in figure 1.

This specimen is an obliquely compressed conical structure consisting of five successively larger rings with interconnecting radial and longitudinal structures (figures 1*a,b,d,h,i* and 2*a*). We orient the specimen such that the smallest ring represents the apical end, and the largest ring the abapical end, and we label them as the first (smallest) to the fifth (largest) ring (figures 1*a* and 2*a*). The minimal length from the apical to abapical end (figure 1*i*) is about 570 µm, and the specimen measures about 2.8 mm in width between the two lateral extremities (figure 1*a,g*). Whereas the first ring is almost circular, the remaining four are obliquely compressed into an elliptical shape, but they were likely also circular originally. The first ring is separate from the remaining four larger rings by a gap (figure 1*d*), and is located almost co-planarly at the centre of, or slightly apical to, the second ring (figure 1*a,d*). There are nine lobes on the first ring, 18 lobes on the second ring, 18 corrugations on the third ring and 18 vertebra-like structures on the fourth ring, whereas the fifth ring is belt-like with no surface structures (figure 1*a–f*). There are localized gaps between the third and fourth and between the fourth and fifth rings (figure 1*a,c,e,f)*, but these gaps may be diagenetic in nature (e.g. dislocation related to oblique compression). There are three circlets of radial structures originating from the outer lateral side of the first ring and extending to the inner side of the third ring (figure 1*g,i*). The circlets are alternately stacked on each other, with the first circlet being most apically positioned and the third one being most abapically positioned (figure 1*b,d*). Intercrossing the third to fifth rings are two circlets of longitudinal structures that run perpendicularly to the rings, with the first extending from the third to fifth rings, and the second occurring mainly on the fifth ring (figure 2*a,b*). Some longitudinal structures in the first circlet become more fibrous in textural appearance toward the abapical end (figure 2*b,d,g*). For detailed description of this specimen, refer to the electronic supplementary material.

Micro-CT analysis shows that the specimen is composed of homogeneous minerals, with few discernable microstructures (figure 1*g–l*; electronic supplementary material, movies S1 and S2). The space between the radial structures is filled with material of low X-ray attenuation and forms grey stripes to denote the boundary between the radial structures (figure 1*g*,*j*,*k*). The grey stripes extend between the first and third rings, and finally disappear around the middle of the third ring (figure 1*l*), indicating that the radial structures may be attached on the inner side of the third ring. This is also supported by the third ‘circlet’ of a single radial structure, which extends abapically to the inner side of the third ring (figure 1*g*,*i*). The grooves or depressions between the adjacent lobes, corrugations and vertebra-like structures on the first, second, third and fourth rings do not incise into the rings, implying that they are integrated structures rather than aggregates of separate subunits.

**Figure 3.**
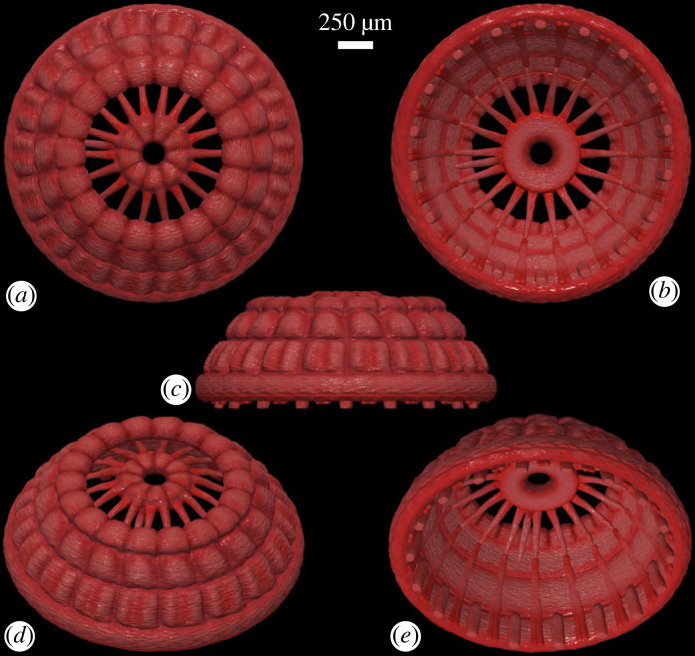
Reconstruction of specimen NIGP179459. (*a*) Apical view, (*b*) abapical view, (*c*) lateral view, (*d*) apical–lateral view, (*e*) abapical–lateral view. Scale bar applies to all images.

An artistic reconstruction of NIGP179459 is depicted in figure 3. The second to fifth rings are coaxially stacked and constitute an apically truncated cone, with hexaradially arranged internal longitudinal structures.

### (b) Nanocrystals of NIGP179459

A closer look at the surface of NIGP179459 under SEM reveals that the specimen is composed of nanocrystals of variable sizes (electronic supplementary material, figure S2). The smallest nanocrystals (150–850 nm in length and 85–310 nm in width) occur on the rings (electronic supplementary material, figure S2c,e,g), and the largest (195–875 nm in length and 86–360 nm in width) on the second circlet of longitudinal structures (electronic supplementary material, figure S2h,i). The crystals are randomly oriented except those on the second circlet of longitudinal structures (electronic supplementary material, figure S2h,i), which appear to be more organized or tessellated. In places, there are tiny holes (1–4 µm in diameter) leading to the interior of the rings, and they are surrounded by radially arranged nanocrystals (electronic supplementary material, figure S2b,d,e,f).

### (c) Other specimens

In addition to NIGP179459, we recovered two further, but more fragmented specimens, NIGP179460 (electronic supplementary material, figure S3a,b) and NIGP179461 (electronic supplementary material, figure S3c,d). These two specimens are similar in size and morphology, and both have a single ring, as well as rope-like radial structures. The number of the radial structures is unclear because some are missing and some are heavily cemented with minerals. In general morphology, these two specimens resemble the apical part of NIGP179459, preserving the first ring and radial structures.

## 4. Discussion

The morphology, topology and mineral fabrics support the notion that specimen NIGP179459 represents a complex of biological tissues rather than an inorganic, diagenetic and/or taphonomic artifact. This specimen is compressed to a certain degree, implying that it was pliable when the organism was alive. The randomly oriented nanocrystals (electronic supplementary material, figure S2) indicate that the specimen was fossilized through impregnation with calcium phosphate, because phosphatic impregnation tends to result in randomly oriented nanocrystals [18]. Exceptions are the tessellated and radially arranged nanocrystals (electronic supplementary material, figure S2d,e,f,i), which may have nucleated on organic substrates.

Based on the stacking arrangement and the inferred pliability of the rings and radial and longitudinal structures, specimen NIGP179459 is interpreted as fossilized musculature. Some longitudinal structures in the first circlet exhibit a fibrous texture toward the abapical end (figure 2*d*,*g*), indicating that they represent myofibrils and thus corroborating the musculature interpretation. Fibrous structures have also been reported in other exceptionally preserved fossils [19–21], and these are all interpreted as myofibrils. Other interpretations for specimen NIGP179459 (e.g. that it comprises an exoskeleton, epidermis or neural tissues) seem unlikely.

The first ring and the remaining four rings may represent two different groups of muscles, not only because the first ring is evidently smaller than the second ring (485 µm versus 1.43 mm in diameter), but also because the first ring is situated almost at the co-planar center of, and has a significant gap from, the second ring. We argue that, even if the muscles described above from specimen NIGP179459 may have been taphonomically shrunken, the topology and relative position of these muscles would remain unaltered. Thus, it is unlikely that the first ring may originally be located apically on the top of the truncated cone formed by the remaining four rings. By contrast, it is likely to regard the co-planar arrangement of the first and second rings as a biological rather than taphonomic feature. If so, this musculature has four groups of muscles: an inner circular, four outer circular, 19 radial, and 36 longitudinal muscles. These muscle groups correspond to the first ring, the remaining four rings, the three circlets of radial structures, and two circlets of longitudinal structures described above. As for the specimens NIGP179460 and NIGP179461, they may be derived from a musculature similar to specimen NIGP179459, and correspond to the first circular muscle and radial muscles, but it is unclear whether these three specimens come from animals of the same species. A microfossil similar to specimen NIGP179459 occurs in the Early Cambrian Age 2 of northern Siberia (fig. 69K of [22]), but its diameter is estimated to be about 660 µm, which is considerably smaller than the specimens studied herein. Muscle tissues may shrink during decay [23], hence, the size of the studied specimens does not necessarily correspond to their original size. Thus, specimens NIGP179459–179461 and the specimen illustrated in [22] may represent broadly similar musculatures despite their different sizes.

Muscles occur widely among eumetazoans, and they have different evolutionary origins [24] in basal animals (e.g. cnidarians and ctenophores) and bilaterians (protostomes and deuterostomes) [2,3]. Cnidarians have epidermal (i.e. ectodermal) and gastrodermal (i.e. endodermal) epitheliomuscular cells [2], and these cells may form longitudinal, circumferential and/or radial fibrils within epidermis, gastrodermis and/or mesoglea [25–27]. Generally, in polyps the epidermal musculature is longitudinal and the gastrodermal musculature is circular, whereas in medusae the coronal muscles in the subumbrella is circular [28]. Specimen NIGP179459 has four groups of muscles in a complex stacking arrangement, and they are fundamentally different from the simple body-wall muscles of cnidarians [25–27]. Ctenophores have true (i.e. non-epithelial) muscle cells that are differentiated into parietal muscles at the base of the epidermis and mesogleal muscles within the mesoglea [29]. Whereas the parietal muscles may form a loose rectangular network of minute fibres [30], the mesogleal muscles generally form loose bundles of giant longitudinal, deep circular and radial muscle fibres [29,31]. Thus, the collective evidence indicates that the specimens studied herein are not derived from cnidarians or ctenophores.

**Figure 4.**
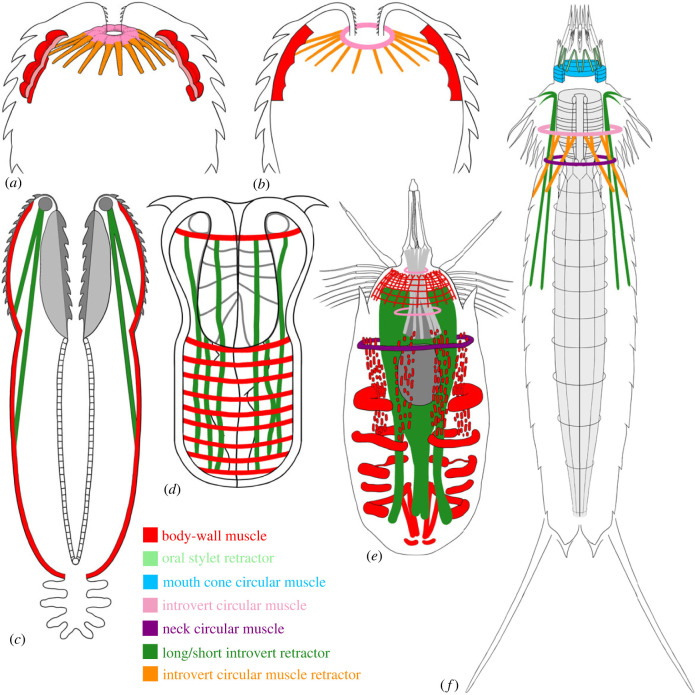
Schematic of scalidophoran musculatures. Introvert everted in all diagrams. (*a,b*) Scalidophorans as represented by specimen NIGP179459, muscles simplified in (*b*); (*c*) adult of *Priapulus* (Priapulida), revised from [40]; (*d*) hatching larva of *Priapulus* (Priapulida), revised from [41]; (*e*) *Nanaloricus* (Loricifera), revised from [42]; (*f*) *Echinoderes* (Kinorhyncha), segmented trunk muscles omitted, revised from [43]. Images not to scale.

Instead, the specimens at hand probably represent musculature of bilaterian animals. Muscle cells of bilaterians are derived from the mesoderm, differentiated into body-wall, visceral and other muscles [2,3]. The body-wall muscles are generally composed of an outer layer of circular and an inner layer of longitudinal muscles [2], sometimes in combination with other muscles, e.g. diagonal ones in platyhelminthes [32]. There may be exceptions to this generalization; for example, nematodes [33], nematomorphs [34], tardigrades [35,36], arthropods [2,37] and some polychaete annelids [38] lack body-wall circular muscles, most likely due to secondary loss. The Early Cambrian lobopodian *Tritonychus* has an outer layer of longitudinal, a middle layer of oblique and an inner layer of circular muscles [21]. Some priapulans may have an additional layer of longitudinal muscles encircling the inner circular and longitudinal muscles in the introvert [39]. The body-wall circular and longitudinal muscles may form a grid surrounding the whole body, for example, in the xenacoelomorph worms [2]; or they may form bundles, each with multiple fibers, e.g. in priapulans (figure 4*c,d*) [39]; or the longitudinal muscles may be arranged into separate bands (e.g. in annelids [44]). The body-wall muscular grid is absent in kinorhynchs (figure 4*f*) [43,45], whereas it is highly elaborate in loriciferans, forming a net-like pattern in the introvert (figure 4*e*) [42,46]. Among the two extant loriciferan taxa, the Pliciloricidae has a body-wall muscular grid within the abdomen, whereas the Nanaloricidae has sets of bilaterally arranged, semicircular abdominal circular muscles and segmented thin longitudinal muscles (figure 4*e*) [42,46]. The digestive tract is also associated with muscles, sometimes arranged as a grid, as in the gut of kinorhynchs (figure 4*f*) [43,47]. Between the body-wall and the visceral muscles, there may be other muscles, such as dorsal–ventral and diagonal muscles in the trunk of kinorhynchs [43], extrinsic and/or intrinsic leg muscles in annelids, onychophorans, tardigrades and arthropods [2], and various retractors in priapulans [40], loriciferans [46], kinorhynchs [45] and sipunculans [48]. Bryozoans have retractors and longitudinal parietal muscles that help to retract the lophophore (tentacle crown) into the trunk, and these muscles are specialized longitudinal muscles [2].

It is possible to further constrain the phylogenetic affinity of the studied fossils based on their similarity to scalidophoran musculatures. The second to fifth rings in the material at hand constitute an apically truncated cone (figure 3), and the 36 evenly distributed longitudinal structures impart a hexaradial symmetry to this cone, inviting a comparison with bilaterian animals whose terminal body parts (head or tail) have a muscular grid of circular and radially arranged longitudinal muscles. Most bilaterians have bilaterally symmetric terminal body parts and bilaterally arranged longitudinal muscles. But there are exceptions. In scalidophorans, for example, the introvert exhibits radial symmetry both externally (i.e. longitudinal rows of scalids are radially disposed) and internally (i.e. longitudinal muscles are radially arranged). More importantly, some fossil and extant scalidophorans exhibit hexaradial symmetry [9,12,49], making them attractive analogues for specimen NIGP179459. Tunicates also have radially arranged longitudinal muscles around the oral and atrial siphons, but these are multifurcated from a much lower number of longitudinal muscles in the middle trunk [50,51]. Panarthropods and lophophorates do not have radially arranged longitudinal muscles around their head or tail [2], thus they are inappropriate models for specimen NIGP179459. Other possibilities, such as everted and disarticulated body parts (e.g. parapodia of annelids, lobopods of tardigrades and onychophorans, and legs of arthropods), are unlikely. The appendages of annelids, tardigrades and onychophorans are controlled by extrinsic leg muscles that are originated from within the body cavity, whereas the legs of arthropods are controlled by extrinsic and intrinsic leg muscles [2]. The arrangement of extrinsic and intrinsic muscles is evidently different from that of specimen NIGP179459. Possible extrinsic leg muscles have been reported from the Early Cambrian lobopodians *Paucipodia* (fig. 5*a* in [52]) and *Tritonychus* (fig. 1*c*,*d* in [21]), and possible extrinsic and intrinsic leg muscles have been reported from the Early Cambrian gilled lobopodian *Pambdelurion* [37], but these are topologically different from the musculature of specimen NIGP179459.

With a possible scalidophoran affinity, we interpret specimen NIGP179459 as the anterior introvert body-wall muscular grid. A complete introvert body-wall muscular grid should be a prolate spheroid in shape, similar to an evaginated introvert (figure 4*c*). However, specimen NIGP179459 is overall hemispheroidal in shape, thus it may represent only the anterior part of the introvert, with the posterior part missing. Since a similar introvert body-wall muscular grid is present in priapulans [40,53] but absent in loriciferans [42,46] and kinorhynchs [43,45], specimen NIGP179459 may belong to the priapulans. But considering that a priapulan-like introvert may have characterized the last common ancestor of the Scalidophora [3,54], the introvert body-wall muscular grid may represent a scalidophoran feature that was lost in crown-group kinorhynchs and loriciferans. The condition in ancestral ecdysozoans is unclear, but the last common ancestor of the Ecdysozoa probably lacked a radially arranged introvert, as inferred from two possible ancestral ecdysozoans: *Acosmia* [54] and *Saccorhytus* [10]. Furthermore, if the Nematoida represents an intermediate evolutionary grade between the Scalidophora and the Panarthropoda [5,55,56], then a priapulan-like introvert may have been inherited in stem-nematoids and stem-panarthropods, but lost in their crown groups. Therefore, it is more conservative to place specimen NIGP179459 in the total-group Scalidophora.

The grid-like pattern in priapulans and in specimen NIGP179459 is different from the net-like pattern of muscles in loriciferans [43]. Whereas the longitudinal muscles of loriciferans may have a bifurcated anterior extremity [42], that is not the case in specimen NIGP179459. Loriciferans have a mouth cone and sets of mouth cone retractors and buccal tube retractors, and their spinoscalids are associated with intrinsic muscles [42]. However, these muscles are absent in specimen NIGP179459, implying that its host animal lacked a mouth cone and that its scalids lacked intrinsic muscles, casting doubt on a loriciferan affinity. Kinorhynchs also have a mouth cone associated with mouth cone circular muscles and oral stylet muscles, but they lack a body-wall muscular grid [43]. Hence, it is unlikely that specimen NIGP179459 belongs to either loriciferans or kinorhynchs.

The scalidophoran affinity of our specimens is further supported by the first ring and radial structures. Guided by modern scalidophorans, we interpret the first ring as the introvert circular muscle, and the radial structures as introvert circular muscle retractors (figure 4*a,b*). Similar circular muscles also occur in other body parts, such as the mouth cone circular muscles of kinorhynchs (figure 4*f*) [43,57,58], and the neck circular muscles of kinorhynchs (figure 4*f*) [43,57,58] and loriciferans (figure 4*e*) [42]. However, whereas the kinorhynch mouth cone circular muscles have oral stylet retractor muscles inserting at their anterior margin, the kinorhynch and loriciferan neck circular muscles have no retractors. The introvert circular muscles (or introvert ring muscles) are exclusive to modern scalidophorans. For example, some adult loriciferans (figure 4*e*) have an anterior and a posterior introvert circular muscle, some larval loriciferans have a single anterior circular muscle [42], and some kinorhynchs (figure 4*f*) have one or more (typically 1–6) introvert circular muscles [43,57,58]. Adult priapulans lack introvert circular muscles (figure 4*c*) [39,40,53,59–61], but their hatching larvae may have a single body-wall circular muscle in the introvert (figure 4*d*; also figure 3*i*,*i*′, *j*,*j*′ in [41]). Thus, the introvert circular muscle may also be an autapomorphic feature of the Scalidophora, and its presence in specimen NIGP179459 suggests a scalidophoran affinity.

Whereas the introvert circular muscle retractors [43] (or introvert short retractors [57,58]) of variable numbers (typically 12–16) occur in different kinorhynchs (figure 4*f*), loriciferans (figure 4*e*) have no introvert circular muscle retractors [42]. Adult priapulans lack introvert circular muscles and thus have no retractors, and their (short and long) introvert retractors insert anteriorly in the vicinity of the circumpharyngeal nerve ring (figure 4*c*) [53,59–62]. However, their hatching larvae may have a single body-wall circular muscle with long retractors, functioning as an introvert circular muscle with retractors (figure 4*d*) [41]. Some Cambrian priapulans are also preserved with possible short and long introvert retractors but without introvert circular muscles (e.g. *Ottoia prolifica*, *Selkirkia columbia* [63], *Xystoscolex* [64] and *Eximipriapulus globocaudatus* [65]). Thus, introvert circular muscle retractors may also be an autapomorphic feature of the Scalidophora. Again, the conditions in ancestral ecdysozoans, stem-nematoids and stem-panarthropods are unclear. Therefore, introvert circular muscle and introvert circular muscle retractors may have characterized the last common ancestor of the Scalidophora, consistent with the total-group scalidophoran interpretation for specimen NIGP179459 (electronic supplementary material, figure S4).

Scalidophorans have different degrees of introvert invagination, varying from complete to partial invagination, and this is controlled by the long introvert retractors. Long introvert retractors are common in priapulans (figure 4*c,d*) [40,53,59–61], kinorhynchs (figure 4*f*) [43,57,58], and loriciferans (figure 4*e*) [42], implying that they can retract their introvert completely into the trunk. Possible stem-priapulan *Ottoia* and *Selkirkia* from the middle Cambrian Burgess Shale biota have various short and long introvert retractors, and thus may have been able to completely retract their introvert [63]. However, specimen NIGP179459 seems to lack long introvert retractors, and its introvert circular muscle appears to be located at the anteriormost margin of the introvert, with very short retractors inserting on the anterior rather than posterior part of the introvert body-wall muscles. Thus the host animals bearing NIGP179459 probably had relatively limited ability to retract the introvert.

Cycloneuralians are abundant and diverse in the fossiliferous bed of the Fortunian Zhangjiagou section (electronic supplementary material, figure S1) [9,12,15] that yielded the material studied herein. Those cycloneuralians are proposed to be early scalidophorans [9,12], which implies that they should have body-wall circular muscles, although such are not preserved. Their introvert scalids exhibit different arrangement patterns, e.g. hexaradial symmetry in *Eopriapulites* [9] and *Shanscolex* [12] and irregular in *Qinscolex* [12]. The circular and longitudinal muscle bundles of some modern priapulans may accord to the rings and rows of introvert scalids [59], and the circular muscles in the net-like muscles of some modern loriciferans may correspond in position to the rings of scalids and attach to the base of each scalid [66]. If these anatomical correlations are applicable to specimen NIGP179459, the host scalidophoran may have at least four circlets of introvert scalids that are arranged into 36 longitudinal rows, exhibiting a hexaradial symmetry. The lobes, corrugations and vertebra-like structures on the second to fourth rings (figure 1*a*) may correspond to the thickened inner surface of the scalids. Whereas an introvert with hexaradially symmetrical scalids may have characterized the last common ancestor of the Ecdysozoa, an introvert with pentaradially symmetrical scalids is restricted to most fossil and modern priapulans and kinorhynchs [49]. Thus, the introvert of *Eopriapulites* [9], *Shanscolex* [12] and the host animal of specimen NIGP179459, with hexaradially arranged scalids, may just be a plesiomorphic feature inherited from the last common ancestor of the Ecdysozoa.

Experimental taphonomy on ecdysozoans reveals that labile tissues such as epidermis, muscles and nerve tissues are among the first to decay after death, leaving only decay-resistant cuticular structures, explaining why labile tissues are rarely preserved as fossils [67–69]. However, a balance between autolysis and microbial activities may facilitate the preservation of the labile tissues [23,70], such as possible nerve tissues [71–75], the cardiovascular system [76] and muscles [37,52,63–65] of ecdysozoans in the Burgess Shale-type Lagerstätten. In the Orsten-type Lagerstätten, taphonomic biases also selectively preserve cuticular structures [13]. However, rare preservation of nerve and muscle tissues occurs (e.g. a possible pharyngeal nerve ring in an embryo of the scalidophoran *Markuelia* [77], and muscle tissues in pentastomids [78], crustaceans [79], olivooid cnidarians [20] and a lobopodian [21]). The present study provides another example of fossilized musculature through three-dimensional phosphatization. It is unclear why the musculatures found in this study are preserved in isolation while in other instances the labile tissues (muscles and nerve tissues) are preserved together with the host animals [20,21,77–79]. One possibility may be that the post-mortem expulsion of gut contents provides a concentration of phosphorus and microbes leading to the preferential phosphatization of muscle tissues. This mechanism is supported by experimental taphonomy [23] and has been observed in several Burgess Shale-type fossils [37,52,63–65]. The fragmentary and tissue-selective preservation of NIGP179459 makes it difficult to constrain its phylogenetic affinity, although comparison with musculatures of living animals does help us to associate this fossil with scalidophorans and possibly priapulans.

In conclusion, the specimens at hand may represent part of the introvert musculature of scalidophorans and possibly priapulans from the Early Fortunian, and this musculature is inferred to have facilitated the inversion of the introvert, locomotion and feeding. Unlike previous analyses based exclusively on cuticular structures [80–82], this work underscores the significance of internal soft anatomy in resolving the affinities of the Cambrian cycloneuralians, and it adds to the remarkable diversity of the Cambrian scalidophorans [83] and underlines the significance of the Orsten-type preservation [13].

## Data Availability

The raw micro-CT data generated in this study have been deposited in Science Data Bank at https://doi.org/10.57760/sciencedb.11228 [17]. Supplementary material is available online [84].
